# Does moderate-to-high intensity Nordic walking improve functional capacity and pain in fibromyalgia? A prospective randomized controlled trial

**DOI:** 10.1186/ar3159

**Published:** 2010-10-13

**Authors:** Kaisa Mannerkorpi, Lena Nordeman, Åsa Cider, Gunilla Jonsson

**Affiliations:** 1 Department of Rheumatology and Inflammation Research, Institute of Medicine, Sahlgrenska Academy, University of Gothenburg, Guldhedsgatan 10, Box 480, 40530 Göteborg, Sweden; 2 Physiotherapy and Occupational Therapy, Sahlgrenska University Hospital, 41345 Göteborg, Sweden; 3 Sahlgrenska School of Public Health and Community Medicine, Institute of Medicine, Sahlgrenska Academy, University of Gothenburg, Box 454, 40530 Göteborg, Sweden; 4 Research and Development Unit in Primary Health Care Södra Älvsborg, Sven Eriksonplatsen 4, 50338 Borås, Sweden; 5 Department of Clinical Neuroscience and Rehabilitation/Physiotherapy, Institute of Neuroscience and Physiology, Box 430, 40530 Göteborg, Sweden; 6 Primary Health Care Uddevalla, Rosenhäll, Sunnanvindsgatan 8, 45160 Uddevalla, Sweden

## Abstract

**Introduction:**

The objective of this study was to investigate the effects of moderate-to-high intensity Nordic walking (NW) on functional capacity and pain in fibromyalgia (FM).

**Methods:**

A total of 67 women with FM were recruited to the study and randomized either to moderate-to-high intensity Nordic Walking (*n *= 34, age 48 ± 7.8 years) or to a control group engaging in supervised low-intensity walking (LIW, *n *= 33, age 50 ± 7.6 years). Primary outcomes were the six-minute walk test (6MWT) and the Fibromyalgia Impact Questionnaire Pain scale (FIQ Pain). Secondary outcomes were: exercise heart rate in a submaximal ergometer bicycle test, the FIQ Physical (activity limitations) and the FIQ total score.

**Results:**

A total of 58 patients completed the post-test. Significantly greater improvement in the 6MWT was found in the NW group (*P *= 0.009), as compared with the LIW group. No between-group difference was found for the FIQ Pain (*P *= 0.626). A significantly larger decrease in exercise heart rate (*P *= 0.020) and significantly improved scores on the FIQ Physical (*P *= 0.027) were found in the NW group as compared with the LIW group. No between-group difference was found for the change in the FIQ total. The effect sizes were moderate for the above mentioned outcomes.

**Conclusions:**

Moderate-to-high intensity aerobic exercise by means of Nordic walking twice a week for 15 weeks was found to be a feasible mode of exercise, resulting in improved functional capacity and a decreased level of activity limitations. Pain severity did not change over time during the exercise period.

**Trial registration:**

Clinicaltrials.gov identifier NCT00643006.

## Introduction

Fibromyalgia (FM) is a non-inflammatory rheumatic disorder characterized by widespread long-lasting pain, fatigue, distress and difficulties in carrying out daily activities. The prevalence of FM ranges from 1% to 3% in the general population, increasing with age and female sex [1]. Criteria for FM include a history of long-lasting widespread pain and pain in 11 of the total of 18 tender points examined at manual palpation [2]. Aberrant physiological pain-processing mechanisms, together with psychological and environmental factors, interact in the development and maintenance of FM.

Pain, fatigue, distress and lack of knowledge about the reasons for pain often lead to a decrease in the level of physical activity and to impaired body functions. Body functions are usually impaired in FM [3] and are associated with limitations in daily activities [4]. Low-intensity exercise, adjusted to the limitations of the patient, has been found to be safe in FM, and is suggested to be an important part of treatment in patients with FM. Walking of low-to-moderate intensity has been found to be safe in FM and to improve function [5,6] and quality of life [7] among FM patients. Exercise in a temperate pool of low-to-moderate intensity has shown improvements in walking ability [8-11], symptoms [10-13] and distress [10,12-14] in sedentary patients with FM.

Some studies have shown that exercise of moderate-to-high intensity improves physical function and pain in FM [15], while others have reported that aggravation of pain [16] or fear of it [17] hindered patients from completing a planned exercise protocol and improving functional capacity. Nordic walking (NW) is a mode of exercise that has gained popularity in Northern Europe in recent years. NW is walking using poles, which implies that muscles in the upper body are also activated and that the length of steps can be increased, which results in a faster gait as compared to walking without poles. Because of the frequent impairments in the function of the upper and lower extremities in patients with FM [3], it was unclear whether NW might be a feasible mode of exercise for this population. To our knowledge, no previous reports have described NW in FM. The aim of this study was to investigate whether moderate-to-high intensity NW could be a feasible exercise in patients with FM and whether it could improve functional capacity and pain in these patients. Our primary hypothesis was thus that moderate-to-high intensity NW, conducted twice a week for 20 minutes for a period of 15 weeks would improve body functions and alleviate pain.

## Materials and methods

### Study design

A randomized controlled trial (RCT) compared the effects of two 15-week exercise programs. The experimental intervention was a supervised moderate-to-high intensity NW program twice a week for 20 minutes, while the active comparator was a supervised low-intensity walking (LIW) program once a week. Trial registration: ClinicalTrials.gov identifier NCT00643006.

The primary outcomes were the six-minute walk test (6MWT) and the Fibromyalgia Impact Questionnaire pain scale (FIQ Pain). Our secondary hypothesis was that the intervention would improve aerobic capacity, overall health status and activity limitations, the two latter variables assessed by the FIQ total score and the FIQ Physical. The exploratory outcomes included a multidimensional questionnaire concerning fatigue.

Criteria for inclusion: Women aged 20 to 60 years with fibromyalgia, defined by the ACR 1990 criteria [2]: a history of long-lasting generalized pain and pain in at least 11 of 18 tender points examined by manual palpation, an ablility to manage a bicycle test at 50 watts or more, and interest in exercising outdoors twice a week for 15 weeks.

Criteria for exclusion: Patients not speaking or reading Swedish, other severe somatic or psychiatric disease, ongoing or planned physical therapy, including exercise, and an inability to accept times for planned exercise sessions.

### Recruitment, examinations and randomization

**Figure 1:**
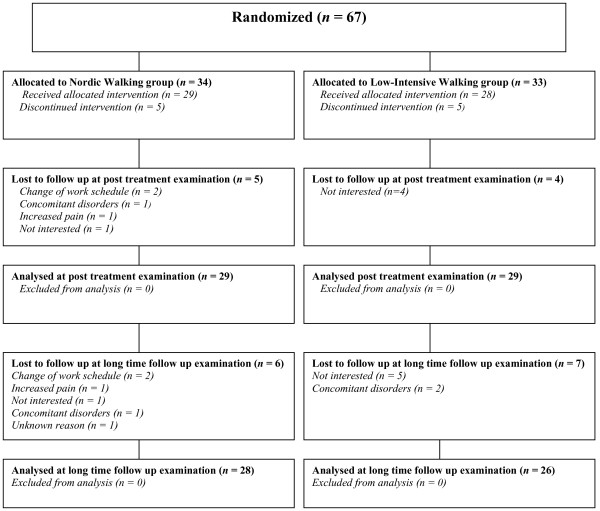
**Participants' flow**.

Female patients were consecutively recruited through newspaper advertisements, at health care centres in West Sweden or from participation in an earlier study. Two hundred and thirty-nine patients were assessed for eligibility. Fifty-four patients did not fulfill the inclusion criteria (other severe disorder *n *= 27, not fulfilling criteria for FM *n *= 16, age >65 years *n *= 6, planned operation *n *= 5). Twenty-four were excluded due to treatment in progress. Seventy-two declined to participate for time restrictions (*n *= 47), negative experiences of NW (*n *= 4), family commitments (*n *= 2), not interested (*n *= 16) or unknown reasons (*n *= 3). Twenty-two could not be contacted. A total of 67 was randomized to the study. Figure 1 shows the participants' flow and reasons for being lost to follow-up.

The screening examination included verification of the ACR 1990 criteria for FM, including a pain localisation sheet [18], a standardized interview and examination of tender points [2]. Patients were then referred to a baseline examination that included a battery of questionnaires, the 6MWT and a submaximal ergometer bicycle test. Body weight and height were measured in light clothing without shoes, and BMI was calculated. Sixty-seven patients completed the baseline examination, where single-blinded examiners did not know the group to which patient would be randomized. After completion of the baseline test the patients were randomized by persons not involved in the examination. Randomization was conducted by using concealed envelopes prepared by the statistician. The numbered envelope was opened together with the patient, after which the patient was informed about the group to which she had been randomized. A total of 34 patients was randomized to NW and 33 to LIW. All patients were invited to a 16-week post-test and a six-month follow-up examination. The patients were advised to continue their baseline medical treatment with no change throughout the 15-week study period.

### Intervention

The study was conducted in parks and forests with flat areas and small hills in one larger (*n *= 32) and two smaller (*n *= 14; *n *= 21) cities in Western Sweden. Each exercise session lasted 40 to 45 minutes, in addition to a five-minute stretching period. Groups of 7 to 15 patients were supervised by two leaders at each session; there were in all 13 exercise leaders, who were physical therapists, physical therapy students or trained exercise leaders.

### Intervention group

Patients randomized to NW participated in supervised exercise sessions twice a week for 15 weeks. The target was to achieve 20 minutes of moderate-to-high intensity exercise. Exercise intensity was based on the subjective perception of exertion, and the patients were instructed as to how to rate exertion on the Borg´s Rating of Perceived Exertion (RPE) scale ranging from 6 to 20 [19]. RPE <12 is considered to correspond to <40% of the maximal heart rate, while 12 to 13 (moderate) corresponds to 40 to 60% and 14 to 16 (heavy) to 60 to 85% of the maximal heart rate [20]. The groups started with light exercise for 10 minutes, ranging from 9 (very light) to 11 (fairly light) on the RPE scale, after which they performed two-minute intervals of moderate-to-high intensity exercise, defined as exertion ranging from 13 to 15 on the RPE scale, alternated with two-minute low-intensity exercise, defined as 10 to 11 on the RPE scale. This means that the participants walked at different speeds in small groups, and the leaders alternated between them to provide individual instruction.

### Control group

Patients randomized to the LIW participated in supervised exercise sessions once a week for 15 weeks. The groups walked at a low intensity level, ranging from 9 (very light) to 11 (fairly light) on the RPE scale.

### Ethics

The study was approved by the ethics committee of Gothenburg University. Written and verbal information was given to all patients and written consent was obtained from all patients. The patients were informed that post-exercise pain could be felt during the first weeks of the exercise program.

### Primary outcomes

For the six-minute walk test (6MWT) the patient was instructed to walk as quickly as possible but not to run. The distance covered was measured in meters [21].

The FIQ Pain is a subscale (0 to 100) of the Fibromyalgia Impact Questionnaire (FIQ), a self-administered questionnaire that assesses health status [22].

### Secondary outcomes

The FIQ Physical (0 to 100) is a subscale of the FIQ that assesses activity limitations. The FIQ total score assesses health status (0 to 100) [22].

Exercise heart rate was measured with a submaximal exercise test on a cycle ergometer (Monark 828E, Varberg, Sweden) with unaltered exercise intensity at baseline and post-test [23]. The selected exercise intensity was either 50 or 75 watts, defined after a structured interview concerning the patient´s physical activity level during the most recent month. The test lasted for six minutes and the pedal frequency was 60 rpm. Heart rate was recorded every minute using telemetry (Polar Electro OY Kempele, Finland), the rate of perceived exertion was assessed on Borg´s Rating of Perceived Exertion (RPE) scale and blood pressure was measured every second minute with a sphygmomanometer. The exercise heart rate was defined as the mean heart value during the fifth and the sixth minute. The subjects were asked to refrain from tobacco, caffeine and food for two hours before the test [24] and from carrying out heavy exercise the day before the visit to the test station. Diurnal variations were taken into consideration by attempting to book the patient at the same time of day at both tests.

### Exploratory outcomes

The Multidimensional Fatigue Inventory (MFI-20) is a self-administered questionnaire that contains 20 statements about fatigue building five subscales ranging from 4 to 20 [25].

### Background variables

The Leisure Time Physical Activity Instrument (LTPAI) assesses the amount of physical activity during a typical week. The total score is the sum of the hours [26]. The Hospital Anxiety and Depression Scale (HADS) builds two subscales: HADS-A for anxiety (0 to 21) and HADS-D (0 to 21) for depression [27]. A cut-off score of eight is suggested to indicate possible anxiety or depression. Muscle tenderness was examined using an algometer (Somedic Production AB, Sollentuna, Sweden), measured in kPa/second [28].

Local pain score (0 to 10) was assessed as the mean value of weekly ratings of pain in the upper and lower extremities recorded in an exercise diary.

### Statistics

On the basis of the results of a previous exercise study [10], we hypothesised that a significant between-group difference for the 6MWT would be found in a sample of 58 patients. Descriptive data are presented as mean, standard deviation (SD), standard error of mean (SE) or the number and percentage. Outcomes were evaluated according to intent-to-treat (ITT) design.

The Mann-Whitney U-test was used to analyse the differences in continuous variables between the two groups. The mean of baseline and post-test values was calculated for each individual and these means were used in group comparisons. Fischer's exact test was used for dichotomous variables and the Mantel-Haenszel chi-two test was used for ordinal categorical variables. Wilcoxon's signed-rank test was used for comparisons of continuous variables within groups, and McNemar´s test was used for dichotomous variables. The Kruskal-Wallis test was used for comparison of the 6MWT in the three sites.

The effect size was calculated for variables that showed a significant change. The effect size for between-group variables was calculated by dividing the mean difference between change from baseline to post-test in the intervention group and the control group by the pooled SD for change. The mean change from baseline to the follow-up test was divided by the SD at baseline to calculate the effect size for follow-up variables. An effect size of 0.20 to 0.50 was considered small, and an effect size >0.50 was considered moderate [29].

Relationships between the variables were examined by Spearman's correlation coefficient. Multivariate stepwise regression analysis was used to study predictors for change.

## Results

### Study population

No significant baseline differences for age, symptom duration, tender point count, pain localisation or pharmacological treatment were found between the NW (*n *= 34) and LIW (*n *= 33) groups, see Table 1. No significant baseline differences were found for body functions, assessed by the 6MWT, the LTPAI, work status, the HAD-D or the education level between the two groups. The HAD-A was found to be significantly higher in the NW (*P *= 0.040) than the LIW group, see Table 1.

**Table 1:** Patient characteristics at baseline in the two groups

	Nordic Walking *n *= 34	Low intensive Walking *n *= 33	
	**Mean **± **SD**	**Mean **± **SD**	*P*-value
Age, years	48 ± 7.8	50 ± 7.6	0.189
Symptom duration, years	11 ± 5.4	12 ± 5.3	0.436
Pain localisation, 0 to 18	14 ± 3.1	14 ± 2.7	0.548
TP, n	15 ± 1.8	15 ± 2.2	0.554
Algometer, kPa/sec	196 ± 56.0	210 ± 64.6	0.437
LTPAI, h	5.6 ± 4.6	4.8 ± 4.3	0.270
BMI	28 ± 4.0	28 ± 4.5	0.990
HAD-A	9.8 ± 5.3	7.2 ± 4.2	0.040
HAD-D	7.6 ± 4.3	6.8 ± 3.9	0.372

	**n (%)**	**n (%)**	
*Pharmacological treatment*			
Analgesics, yes	28 (82%)	27 (82%)	1.000
Antidepressants/sedatives, yes	24 (70%)	21 (64%)	0.433
Beta blocks, yes	4 (12%)	3 (9%)	0.517
*Education, years*			
≤ 9	3 (9%)	10 (30%)	
10 to 12	18 (53%)	11 (33%)	
>12	13 (38%)	12 (36%)	1.195
*Work status*			
100 to 80%	8 (24%)	4 (12%)	
79 to 50%	8 (24%)	11 (33%)	
<50%	3 (9%)	4 (12%)	
Not working	15 (44%)	14 (42%)	0.696
*Sick-leave*			
None	31 (91%)	30 (91%)	
25 to 75%	1 (3%)	3 (9%)	
100%	2 (6%)	0 (0%)	0.574
*Sick- or disability pension*			
None	13 (38%)	7 (21%)	
25 to 75%	8 (24%)	13 (39%)	
100%	13 (38%)	13 (40%)	0.370

*P*-values for the differences between the groups are given.

TP, tender point, LTPAI, Leisure Time Physical Activity Instrument, BMI, body mass index, HAD-A, Hospital Anxiety and Depression - Anxiety, HAD-D, Hospital Anxiety and Depression - Depression, SD, standard deviation.

All patients were invited to a post-test according to an intent-to-treat design, and 58 patients (87%) of the total sample completed the test. The median attendance rate at the exercise sessions was 62% (range 0 to 100%) in the NW group and 50% (range 0 to 93%) in the LIW group. Internal missing values in the NW group were found for one person, who refused to walk due to plantar fasciitis, in the 6MWT and in a protocol in the MFI. Internal missing data in the LIW group were found for the FIQ in a protocol and for the MFI in two protocols.

No significant change over time was found for the LTPAI in the NW (*P *= 0.09) or the LIW group (*P *= 0.26). The post-test score in the LTPAI in the NW group was 6.3 (SD 4.2, range 1.0 to 19.0), while it was 5.9 (SD 6.4, range 1.0 to 29.0) in the LIW group. Neither was any significant change (*P *= 1.00) found for use of analgetics, as 76% in the NW and 79% in the LIW group reported use of analgetics at the post-test. No significant change (*P *= 0.50; *P *= 1.00) was found for use of antidepressants or sedatives over time, as intake was reported by 76% in the NW and 68% in the LIW group at the post-test. See Table 1 for baseline values.

The mean value of the local pain score at Week 2 was 6.2 (SD 2.00, range 1.9 to 9.3) in the NW group and 6.7 (SD, 1.8, range 4.0 to 9.3) in the LIW group. The post-test local pain score in the NW group was 5.7 (SD 2.2, range 1.3 to 8.7) and 6.4 (SD 2.2, range 1.7 to 10.0) in the LIW group. No significant change in the local pain score was found over time within the groups (NW *P *= 0.37; LIW 0.23) or between the groups.

### Primary outcomes

A significantly greater improvement (*P *= 0.009) was found in the change in the 6MWT in the NW group (37.7, SD 41.8) as compared to the LIW group (8.6, SD 42.2) (Table 2). The effect size of the 6MWT for the intervention group compared with the control group was 0.69. The difference between the groups was maintained when adjusted for HAD-A (*P *= 0.021). No significant baseline differences (*P *= 0.129) were found for the 6MWT at baseline between the three sites. Mean difference of the 6MWT between the two groups at Site 1 was 38.8 (SE 18.4), at Site 2 it was 21 (SE 25.5), and at Site 3 it was 27.9 (SE 15.4).

**Table 2:** Changes from baseline to post-test within and between the intervention group engaging in Nordic Walking and the control group engaging in Low-intensive walking

	Nordic walking (NW)			Low-intensive walking (LIW)			Δ NW vs. Δ LIW
			
	Baseline Mean (SD) *N *= 34	Post test vs. baseline Δ (SD) *N *= 29^a^	*P*-value	Baseline Mean (SD) *N *= 33	Post test vs. baseline Δ (SD) *N *= 29^b^	*P*-value	*P*-value
*Primary Outcomes*							
6MWT, meter	525 (71.1)	37.7 (41.8)	**<0.001**	522 (56.1)	8.6 (42.2)	0.105	**0.009**
FIQ Pain, score	63.9 (21.2)	-4.0 (14.5)	0.190	71.5 (20.7)	-5.3 (16.3)	0.065	0.626
*Secondary outcomes*							
Exercise heart rate at ergometer test, beats/minute	124.5(17.1)	-8.9 (12.8)	**0.001**	121.1 (12.2)	-3.1 (9.6)	0.079	**0.020**
FIQ Physical, score	42.8 (20.5)	-7.9 (12.6)	**0.004**	41.9 (23.35)	1.3(15.6)	0.929	**0.027**
FIQ Total, score	61.7 (18.3)	-4.8(12.3)	**0.048**	62.9 (18.6)	1.9(14.2)	0.374	0.064
*Explorative variables* *MFI, score*							
General Fatigue	17.0 (3.1)	-0.9 (2.9)	0.091	17.3 (3.1)	-0.1 (2.7)	0.972	0.116
Physical Fatigue	16.2 (3.5)	-0.5 (2.8)	0.319	17.2 (3.5)	-1.0 (3.6)	0.280	0.932
Reduced Activity	14.5 (4.5)	-0.2 (2.8)	0.476	15.6 (4.4)	-0.5 (4.0)	0.194	1.000
Reduced Motivation	11.7 (4.3)	-1.0 (3.0)	0.072	10.3 (4.4)	0.9 (3.2)	0.287	**0.031**
Mental Fatigue	14.9 (3.5)	0.1 (2.5)	0.608	15.1 (3.9)	-0.4 (2.6)	0.461	0.336

Mean values, standard deviations (SD) and *P*-values for changes within and between the groups are given.

^a^The 6MWT was missing in a protocol, and the MFI scores in another. ^b^The FIQ scores were missing in a protocol and the MFI scores in two protocols. 6MWT, Six-Minute Walk Test, FIQ, Fibromyalgia Impact Questionnaire, MFI, Multidimensional Fatigue Inventory.

The FIQ Pain did not change significantly (*P *= 0.626) in the between-group analysis of the NW and LIW groups.

### Secondary outcomes

A significantly higher improvement (*P *= 0.027) was found for the change in the FIQ Physical (-7.9, SD 12.6) in the NW group as compared with the control group (-1.3, SD 15.6) (Table 2). The effect size of the FIQ Physical in the intervention group as compared with the control group was 0.64. The FIQ Total score did not change significantly (*P *= 0.064) in the between-group analysis of NW and LIW.

A significantly larger decrease (*P *= 0.020) of the heart rate in the submaximal ergometer test was found in the NW group (-8.9, SD 12.8) as compared with the change in the control group (-3.1, SD 9.6). The effect size of the heart rate for the intervention group compared with the control group was 0.51.

### Exploratory outcomes

A significantly higher improvement (*P *= 0.031) was found for the change in the MFI Reduced Motivation in the NW (-1.0, SD 3.0) as compared with the LIW group (0.9, SD 3.2) (Table 2). The effect size of the FIQ total score in the intervention group as compared with the control group was 0.60.

### Six-month follow-up of NW

A total of 28 patients in the NW group completed the six-month follow-up examination.

Primary outcomes. A significant improvement (*P *= 0.009) was found for the change of the 6MWT (20.9, SD 52.5) in the NW group, the effect size being 0.29. No significant improvement was found for the FIQ Pain (*P *= 0.879).

Secondary outcomes. No significant improvements were found for the FIQ Physical (*P *= 0.542), FIQ total score (*P *= 0.249) or exercise heart rate (*P *= 0.232).

Explorative outcomes. A significant improvement was found for the MFI General Fatigue (-2.1, SD 2.2, *P *<0.001) and the MFI Physical Fatigue (-1.9, SD 2.5, *P *= 0.001), and the effect size was 0.68 for the MFI General Fatigue and 0.54 for the MFI Physical Fatigue. No significant changes were found for the MFI Reduced Activity (*P *= 0.743), the MFI Reduced Motivation (*P *= 0.084), or the MFI Mental Health (*P *= 0.725).

### Six-month follow-up of the LIW

A total of 26 patients in the LIW completed the six-month follow-up.

Primary outcomes. No significant improvement was found for the 6MWT (*P *= 0.603) or the FIQ Pain (*P *= 0.412).

Secondary outcomes. No significant changes were found for the FIQ Physical (*P *= 0.710), FIQ total score (*P *= 0.638) or exercise heart rate (*P *= 0.326).

Exploratory outcomes. A significant improvement (*P *= 0.001) was found for the MFI General Fatigue (-2.3, SD 2.6, *P *<0.001) and the MFI Physical Fatigue (-2.5, SD 3.4, *P *= 0.001). The effect size was 0.74 for the MFI General Fatigue and 0.71 for the MFI Physical Fatigue. No significant changes were found for the MFI Reduced Activity (*P *= 0.794), the MFI Reduced Motivation (*P *= 0.147), or the MFI Mental Health (*P *= 0.513).

### Predictors of change of the 6MWT

Multivariate stepwise regression analysis was used to study possible predictors for change in the 6MWT (Table 3). Age, education level, group randomized to, attendance rate, number of TPs, pain localisation, pain duration, baseline values of the HAD-D, the HAD-A, the FIQ total score, the LTPAI total score and the 6MWT were chosen as independent variables. Three models appeared, and the model showing the largest adjusted R square, which was 0.31, is presented in Table 2. This model indicates that 31% of the variability in the 6MWT was explained by the baseline value of the 6MWT, the number of TPs and the group randomized to, *P *= 0.012.

**Table 3:** Multivariate stepwise regression

Variable	Parameter Estimator	(SE)	*P*-value
Intercept	329.7	(64.13)	
6MWT	-0.30	(0.08)	0.000
TPs	-7.43	(2.85)	0.010
Group	-26.6	(10.14)	0.012

Independent variables are: Age, education, group, attendance rate, number of TPs, pain localisation, pain duration, baseline values of the HAD-A, the HAD-D, the FIQ total score, the LTPAI total score and the 6MWT. The dependent variable is change of the 6MWT.

6MWT, Six-Minute Walk Test; TP, tender point; HAD-A, Hospital Anxiety and Depression - Anxiety; HAD-D, Hospital Anxiety and Depression - Depression; FIQ, Fibromyalgia Impact Questionnaire; LTPAI, Leisure Time Physical Activity Instrument; SE, standard error.

When the baseline value of the 6MWT was eliminated, the only predictor for change of the 6MWT was group (*P *= 0.020). The adjusted R square for that model was 0.08.

## Discussion

Patients with FM that participated in a 15-week supervised Nordic walking program twice a week significantly improved their functional capacity, as assessed by the 6MWT, as compared with the active comparators that participated in low-intensive walking, as hypothesized. This result agrees with the Cochrane review of the effects of exercise in FM [15]. The effect size indicated a moderate improvement of the 6MWT in the NW group as compared with the control group. The improved outcome in the 6MWT corresponded with the reports of the exercise leaders, who described a successive increase in walking speed at the exercise sessions in most of the participants in the NW group.

A multivariate stepwise regression analysis showed that a lower baseline capacity of the 6MWT, a lower number of TPs and participation in NW predicted the improvement in the 6MWT. The number of tender points has previously been related to pain and overall health [30]. The present study found a low association between the number of TPs and FIQ Pain (rho 0.31, *P *= 0.01), the number of pain localisations (rho 0.32, *P *= 0.008) and the FIQ total (rho 0.27, *P *= 0.03). As some patients were fast walkers, with a baseline value of the 6MWT of about 600 meters, the outcome was probably biased by ceiling effects among those participants.

A significantly reduced heart rate was found in a submaximal ergometer test in the NW group after the exercise period, as compared with the change that occurred in the control group. A test with an unaltered exercise intensity was chosen for this study because of the common use of beta blockers and/or anti-depressants in FM, which may affect heart rate, and since there is a known difficulty in FM in increasing the resistance on an ergometer bicycle [16]. The heart rate can also be affected by diurnal variations and use of caffeine or tobacco. Attempts were made to control these factors by booking the patients at the same time of day and asking them not to change their medication during the study period and to avoid their use of tobacco, caffeine and food two hours before the ergometer cycle test. No documentation was made as to the prevalence of smokers; a patient's heart rate was compared only with her own baseline values. Despite the methodological limitations involved with the assessment of heart rate, the results indicate that women with FM may decrease their exercise heart rate by regular moderate-to-high intensity exercise, as suggested by previous studies in the field [31].

The second hypothesis, that exercise of a moderate-to-high intensity would decrease overall pain more than low-intensity walking, was not supported by this study. The severity of pain, rated on the FIQ Pain, did not significantly decrease over time in any of or between the two groups, but high standard deviations indicate a large variation among the participants. The results agree with a recent review of exercise programs for patients with FM, suggesting that most patients do not experience a decrease in pain by aerobic exercise [32].

Activity limitations in daily life (the FIQ Physical) improved significantly in the NW group, as compared to the change in the LIW. Health status (the FIQ Total) improved significantly in the NW group, which agrees with a previous study of exercise in FM [11].

It was expected that there would be an increase in post-exercise pain during the initial phase of the exercise program due to dysfunctions in peripheral and central pain mechanisms in FM [33], and a temporary increase of pain was reported by several patients at exercise sessions, not only participants in the NW group but also participants in the LIW group. Concomitant local musculoskeletal pain conditions in the lower extremities were found to limit an increased walking speed among some patients. Patients who reported disturbing post-exercise pain were advised to reduce their walking speed until such time that they could again increase it. One reason for this was to try to prevent a deterioration of the overall pain condition, which may be induced by peripheral musculoskeletal pain [32,34].

Only one patient interrupted the exercise program due to adverse effects of exercise. She had chronic trochanteritis, which deteriorated after a few exercise sessions. On the group level, the weekly local pain scores did not indicate differences over time between the two groups. Pharmacological treatment did not significantly change from baseline to post-test in any of the groups, but some patients reported that they had temporarily increased their use of analgesics during the first weeks of NW before adaptation to the exercise program.

The follow-up examination indicated that the improvement in the 6MWT was still significant in the NW group six months after the start of the program, although the change between follow-up and baseline was small. Aerobic capacity no longer indicated a significant improvement. These results show that it is difficult for patients with FM to continue exercising at a moderate-to-high intensity level without supervision.

The follow-up examination showed a significant improvement in the MFI General Fatigue and MFI Physical Fatigue, with a moderate effect size in both groups. The results indicate that regular exercise over a longer period of time may decrease fatigue in FM, which is in agreement with a previous study [11]. Alternative explanations for decreased fatigue might be social interactions and support during the exercise sessions, fresh air, or a positive effect of nature.

The overall aim of the NW program was to attain 20 minutes of exercise of moderate-to-high intensity, defined by perceived exertion using an RPE scale [19]. The participants were taught to exercise at two-minute intervals of low- and high-intensity. They walked at different speeds since their baseline walking capacity varied greatly. Some participants managed the predetermined level of exercise well and successively increased their walking speed, even when climbing hills, in comparison with those who found it difficult to comply with the intensity of exercise, referring to pain or other disabilities. The poles were also found to improve stability and balance when walking downhill. The program appeared to have worked well in most participants, as shown by the positive results. Self-reported anxiety did not appear to negatively impact the ability to exercise or the ability to increase the walking speed.

The baseline walking capacity of the participants and its progress was not found to differ between the three sites.

The outcomes were analysed according to an intent-to-treat design, implying that patients that did not attend a session were also invited to participate in a post-test and were investigated accordingly. Eighty-seven percentage of the patients participated in the post-test examination, indicating satisfactory compliance. The median attendance rate was 62% in the NW and 50% in the LIW sessions, a rate that can be expected in patients with severe health problems. Reasons for absence included concomitant disorders, such as the flu, time limitations and pain. As patients were advised to exercise in their own environment if they failed to attend a session, the documented attendance rate might be lower than the actual frequency of outdoor walks during the exercise period.

High-intensity exercise is troublesome for patients with FM. A previous study evaluating the effects of aerobic dance described a high attrition rate and deterioration of symptoms due to exercise-induced pain, not finding any improvement in functional capacity [17]. Another study of aerobic exercise found increased pain in the group engaging in high-intensity exercise on a stationary bicycle when compared to those randomized to low-intensity exercise [16]. Compared to these two modes of exercise, the NW program worked better. A reason may be the use of poles, which facilitates walking and relieves the weight load on the lower extremities. Exercising in short intervals was also found to be a feasible mode for this patient group, as it provided a short rest after each vigorous interval. NW therefore appears to be a viable option for patients with FM wishing to improve their functional capacity without risking a pain flare.

In terms of clinical implications, it appears to be important to determine the patient's baseline capacity before starting to exercise, and to adjust exercise for patients to their baseline condition. If concomitant local pain conditions in the lower extremities hinder faster walking, patients should be recommended to start exercising at a slow speed and shorter walking distance and then to successively increase the speed and distance. Supervision by a health professional who teaches patients to use the poles, helps them to adjust the intensity of their exercise to their limitations and motivates them to increase their walking speed during high intensity intervals is suggested to be valuable for outcomes.

A limitation of the present study is a lack of an untreated control group. Supervised LIW was used to control for improvement through attention, but a third non-treated group should be considered in future studies to evaluate non-specific effects of exercise such as fatigue. As earlier described, the changes in heart rate in an ergometer bicycle test should be interpreted with caution in patients with FM owing to the pharmacological treatment and other factors that may affect heart rate.

## Conclusions

In conclusion, a supervised 15-week NW program designed to alternate between low and moderate-to-high exercise intensity, was found to be a feasible mode of exercise for patients with FM. Most patients tolerated this mode of exercise, and pain severity did not change significantly over time during the exercise period. The participants in the NW program improved their functional capacity and decreased their level of activity limitations compared to active comparators. Thus NW appears to be a viable option for patients with FM who wish to improve their functional capacity by exercise without risking a pain flare.

## Abbreviations

6MWT: six-minute walk test; ACR: American Colleague of Rheumatology; FIQ: Fibromyalgia Impact Questionnaire; FM: fibromyalgia; HADS: Hospital Anxiety and Depression Scale; ITT: intent-to-treat; LIW: low-intensity walking; LTPAI: Leisure Time Physical Activity Instrument; MFI: Multidimensional Fatigue Inventory; NW: Nordic walking; RCT: randomized controlled trial; RPE: Rating of Perceived Exertion; SD: standard deviation; SE: standard error of mean; TP: tender points.

## Competing interests

The authors declare that they have no competing interests.

## Authors' contributions

KM conceived the study, acquired the funding, participated in the design of the study and took responsibility for recruitment, enrollment, statistical analysis and drafting of the manuscript. LN participated in recruitment, enrollment, drafting the manuscript and data collection. ÅC participated in the design of the study, statistical analysis, drafting the manuscript and data collection. GJ participated in recruitment, enrollment, drafting the manuscript and data collection. All authors read and approved the final manuscript.
